# Strabismus management in retinoblastoma survivors

**DOI:** 10.1186/s12886-024-03379-9

**Published:** 2024-03-13

**Authors:** Babak Masoomian, Carol L. Shields, Hamid Riazi Esfahani, Atefeh Khalili, Fariba Ghassemi, Pukhraj Rishi, Mohammad Reza Akbari, Masoud Khorrami-Nejad

**Affiliations:** 1 Ocular Oncology Department, Farabi Eye HospitalFarabi Eye Hospital, Farabi Hospital, Tehran University of Medical Sciences, South Kargar Street, Qazvin square, Tehran, Iran; 2 Pediatric Ophthalmology Department, Farabi Eye Hospital, Tehran University of Medical Sciences, Tehran, Iran; 3 Ocular Oncology Service, Wills Eye Hospital, Thomas Jefferson University, Philadelphia, PA USA; 4 Truhlsen Eye Institute, Omaha, NE USA; 5 School of Rehabilitation, Tehran University of Medical Sciences, Tehran, Iran

**Keywords:** Retinoblastoma, Strabismus, Chemotherapy, Surgery

## Abstract

**Purpose:**

To report the result of strabismus surgery in eye-salvaged retinoblastoma (Rb) patients.

**Methods:**

A retrospective case series including 18 patients with Rb and strabismus who underwent strabismus surgery after completing tumor treatment by a single pediatric ophthalmologist.

**Results:**

A total of 18 patients (10 females and 8 males) were included with a mean age of 13.3 ± 3.0 (range, 2–39) months at the time tumor presentation and 6.0 ± 1.5 (range, 4–9) years at the time of strabismus surgery. Ten (56%) patients had unilateral and 8(44%) had bilateral involvement and the most common worse eye tumor’s group was D (*n* = 11), C (*n* = 4), B (*n* = 2) and E (*n* = 1). Macula was involved by the tumors in 12 (67%) patients. The tumors were managed by intravenous chemotherapy (*n* = 8, 47%), intra-arterial chemotherapy (*n* = 7, 41%) and both (*n* = 3, 17%). After complete treatment, the average time to strabismus surgery was 29.9 ± 20.5 (range, 12–84) months. Except for one, visual acuity was equal or less than 1.0 logMAR (≤ 20/200) in the affected eye. Seven (39%) patients had exotropia, 11(61%) had esotropia (*P* = 0.346) and vertical deviation was found in 8 (48%) cases. The angle of deviation was 42.0 ± 10.4 (range, 30–60) prism diopter (PD) for esotropic and 35.7 ± 7.9 (range, 25–50) PD for exotropic patients (*P* = 0.32) that after surgery significantly decreased to 8.5 ± 5.3 PD in esotropic cases and 5.9 ± 6.7 PD in exotropic cases (*P* < 0.001). The mean follow-up after surgery was 15.2 ± 2.0 (range, 10–24) months, in which, 3 (17%) patients needed a second surgery.

**Conclusion:**

Strabismus surgery in treated Rb is safe and results of the surgeries are acceptable and close to the general population. There was not associated with tumor recurrence or metastasis.

## Introduction

Retinoblastoma (Rb) is the most common primary intraocular malignancy in children and this malignancy has been managed by enucleation (eye removal) for a at least 100 years [1]. Retinoblastoma management has undergone dramatic changes in the last decades and could increase survival rates to more than 90% in developed countries and also high globe salvage rate, according to the grade of tumor burden [1, 2].

Intravenous chemotherapy (IVC), was found to achieve > 90% tumor control when used for International Classification of Retinoblastoma (ICRB) group A to C, and 47% for group D eyes [2]. With the addition of new treatment modalities, such as Intra-arterial chemotherapy (IAC) and Intra-vitreal chemotherapy (IVit), higher tumor control rates are happening [3]. A more recent study by Shields et al. demonstrated that IAC provided remarkable improvement in globe salvage rate over IVC in group D eyes (91% vs. 47%, *p* = 0.004). They also showed a 36% globe salvage rate in group E eyes by IAC [3].

Following the developments, Given the advances in globe-sparing therapy, new concerns are the improvement of functional ocular outcomes including visual acuity, ocular misalignment, and even binocular vision abilities [4–7].

Retinoblastoma patients with a foveal lesion or detached retina, are at risk of developing low vision and strabismus [8]. Strabismus, may persist as the only sign of disease or worsen and develop at a later stage in Rb patients [8]. However, this condition may result in negative psychosocial implications on the parents and patient’s life [14–18]. A limited number of studies have been performed regarding ocular functions after successful tumor treatment whereas, most of them have focused on patients’ visual acuity [4–7].

The present case series aimed to report our surgical experience and the result of strabismus surgery in globe salvaged Rb patients following multimodality treatment with manifest deviation.

## Methods and materials

This retrospective case series study was performed on patients managed in a tertiary referral retinoblastoma center in Farabi eye hospital, Tehran, Iran from February 2019 till October 2021. All patients with bilateral or unilateral involvement that were not enucleated and underwent strabismus surgery were included.

The following data were retrieved from the records: patient age, sex, family history of Rb, laterality of tumor, presenting signs, and clinical variables at presentation. All involved eyes were classified according to the ICRB (International classification of Retinoblastoma) group.

All of the patients received intra-arterial chemotherapy (IAC) and/or intravenous chemotherapy (IVC) and some of them also had adjuvant therapy with cryotherapy, transpupillary thermotherapy (TTT) or Intravitreal chemotherapy (IVit). IVC regimen included vincristine, etoposide, and carboplatin, every three weeks for eight cycles and IAC regimen included topotecan and melphalan for at least three cycles. IVit chemotherapy was done in the case of refractory vitreous seeds to IAC and/or IVC with multiple intravitreal injections of melphalan and/or topotecan.

Patients who did not need further treatments (including chemotherapy or adjuvant therapy) in the last 12 months and whose age was more than four years were included.

The orthoptic evaluation included visual acuity (VA) examination, Krimsky test, cover and uncover test (if possible) at near (1/3 m) and distance (6 m), ocular motility examination, and nystagmus assessment. Visual acuity was assessed using Cardiff acuity cards or Snellen acuity, depending on the child’s age. Snellen VA was converted to logMAR equivalent. Corrected distance visual acuity (CDVA) less than 20/400 was considered as follows: finger count: 2.0 logMAR; hand motion: 2.3 logMAR; light perception: 2.6 logMAR; and no light perception (NLP) = 2.9 logMAR. After doing deviometry, strabismus surgery was performed based on Park’s Table [9].

All surgeries were performed by one surgeon (B.M). The patients were visited at one week, three months, and then for six months intervals after surgery. The surgical success was defined as orthotropia or less than eight prism diopters (PD) over or under correction. Strabismus surgeries were performed for all patients with non-adjustable procedures using direct placement of extraocular muscles. The method of using stitches and surgical techniques were completely similar to non-Rb patients. After strabismus surgery, all patients were examined by an ocular oncologist according to a regular schedule.

The statistical analysis was performed using SPSS version 26.0 software (IBM, Armonk, NY, USA). The Wilcoxon test was used to compare the angle of deviation before and after surgery. Chi-square test was performed to compare the frequency of esotropia and exotropia among Rb patients. All *P*-values less than 0.05 were considered statistically significant.

## Results

There were 18 children (10 females and 8 males) included in this study. The mean age at the time of tumor presentation was 13.3 ± 3.0 (range, 2–39) months. Unilateral and bilateral types of Rb were observed in 10 (56%) and 8 (44%) patients, respectively. The most common worse eye ICRB group was group D (*n* = 11), and the remaining were groups B (*n* = 2), C (*n* = 4), and E (*n* = 1). The macula was involved by the tumors in 12 (67%) of cases. IVC and IAC were the main treatment in which 8 (47%) patients received IAC and 7 (41%) patients received IVC. Three (17%) patients were managed with both treatment modalities. Adjuvant focal therapy, consisting of laser thermoplasty alone or a combination of laser and cryotherapy, was performed for thirteen cases. Six patients underwent IVit chemotherapy for the treatment of vitreous seeds. Of note, no patient had intracranial or distance metastasis. The patient's demographics and tumor treatment features are listed in Table 1.

**Table 1:** Comparative analysis of 18 consecutive patients with strabismus secondary to retinoblastoma. Patients’ demographics and treatment features

No.	Age at tumor presentation (months)	Sex	Laterality	Affected eye tumor group	Macular involvement	Chemotherapy	Intra vitreous chemotherapy	Adjuvant therapy
1	8	F	OS	C	Yes	IAC*1	−	−
2	9	F	OU	D	Yes	IVC*16	−	+
3	10	M	OU	D	Yes	IVC*8	−	+
4	2	M	OU	D	No	IAC*3	−	+
5	20	M	OD	D	Yes	IVC*8	+	+
6	6	M	OU	C	No	IVC*8	−	+
7	20	F	OS	E	Yes	IAC*3/IVC*12	−	+
8	6	F	OU	B	Yes	IVC*8	−	+
9	39	M	OD	D	No	IAC*2	−	−
10	27	F	OS	D	No	IAC*3	+	−
11	24	F	OU	B	Yes	IVC*6	−	+
12	7	M	OS	C	No	IAC*2	−	+
13	11	F	OD	D	Yes	IAC*4/IVC*8	−	+
14	5	F	OU	D	Yes	IVC*8	−	+
15	8	F	OU	C	Yes	IVC*12	+	+
16	12	M	OS	D	No	IAC*2	+	−
17	14	M	OS	D	Yes	IAC*2	+	−
18	12	F	OD	D	yes	IAC*1/IVC*8	+	+

F, female; M, male; IAC, intraarterial chemotherapy; IVC, intravenous chemotherapy; Adjuvant therapy, transpupillary thermotherapy and/or cryotherapy

After completing the antitumor treatment, the average time to strabismus surgery was 29.9 ± 20.5 (range, 12–84) months. The mean age of patients at the time of strabismus surgery was 6.0 ± 1.5 (range, 4–9) years. The mean visual acuity in the affected eye was 1.9 ± 0.7 logMAR (range, 0.2–2.9) and in the non-affected eye was 0.02 ± 0.5 logMAR (range, 0.0-0.2) (*P* < 0.001). Except for one patient (VA = 20/40), all patients had poor vision in the affected eye (less than 1.0 logMAR) (Table 2). Seven (39%) patients had exotropia, and esotropia was observed in 11(61%) cases (*P* = 0.35). The pure horizontal deviation was detected in 9 (50%) and combined horizontal and vertical deviation was found in 9 (50%) cases. Three (17%) patients had hypotropia and hypertropia was observed in 6 (33%) cases, while inferior oblique overaction was the reason for hypertropia in 4 of these 6 cases.

**Table 2:** Comparative analysis of 18 consecutive patients with strabismus secondary to retinoblastoma. Strabismus feature, management and outcome

No.	Age at strabismus surgery (years)	VA (affected eye)	VA (fellow eye)	Time to surgery (months)	Strabismus feature (pd)	IOOA	Type of surgery	Outcome of surgery	Re-operation	f/u (months)
1	4	HM OS	20/20	12	LXT (40) LHT (20)	Yes	R&R(OS) + IO myectomy	20pd ET	Yes	18
2	4	NLP OS	20/20	12	LXT (25) LHOT (15)	No	R&R(OS) FT up+	5pd ET	No	18
3	9	CF OS	20/25	84	LET (40) LHT (12)	No	R&R(OS) FT down+	orthotropia	No	18
4	5	HM OD	20/20	36	RXT (30)	No	R&R(OD)	orthotropia	No	24
5	6	HM OD	20/20	24	RET (30)	No	RMR recess + PF	8pd ET	No	24
6	8	20/200OD	20/25	60	RET (30)	No	RMR recess	8pd ET	No	18
7	8	HM OS	20/20	36	LXT (35) LHOT (14)	No	R&R (OS) FT up+	6pd XT	No	18
8	6	20/200 OS	20/30	12	LET (50)	No	R&R(OS)	6pd XT	No	18
9	7	CF OD	20/20	24	RET (30)	No	RMR recess	6pd ET	No	30
10	6	NLP OS	20/20	12	LET (35)	No	R&R(OS)	8pd ET	No	18
11	5	HM OS	20/30	36	LXT (35)	No	R&R(OS)	4pd ET	No	14
12	4	CF OS	20/20	16	LET (55) LHOT (16)	No	R&R/ (OS) FT up+	8pd ET	No	18
13	7	HM OD	20/20	24	RXT (35) RHT (10)	Yes	R&R(OS) + IO myectomy	6pd ET	No	20
14	6	20/40 OS	20/40	40	ALTET (35) LHT (6)	Yes	R&R(OS) + IO myectomy	20pd XT	Yes	24
15	6	20/200 OD	20/200	12	ALT XT (50)	No	R&R(OD)	orthotropia	No	20
16	5	HM OS	20/20	14	LET (40) LHT (10)	Yes	R&R(OS) + IO myectomy	4pd XT	No	18
17	5	CF OS	20/20	24	LET (40)	No	R&R (OS)	15pd XT	Yes	18
18	7	NLP OS	20/20	60	LET (60) LHT (10)	No	R&R(OS) FT down+	8 pd ET	No	12

HM, hand motion; CF, counting finger; NLP, no light perception; ET, esotropia; XT, exotropia; HT, hypertropia; HOT, hypotropia; ALT, alternate; FT, full tendon; IOOA, inferior oblique overaction; pd, prism diopter; R&R, recess and resection; RMR, right medial rectus; PF, posterior fixation suture

Unilateral constant strabismus was detected in 16 (89%) patients in their affected eye with low visual acuity compared to 2 (11%) patients who had alternate strabismus (*P* < 0.001).

**Fig. 1:**
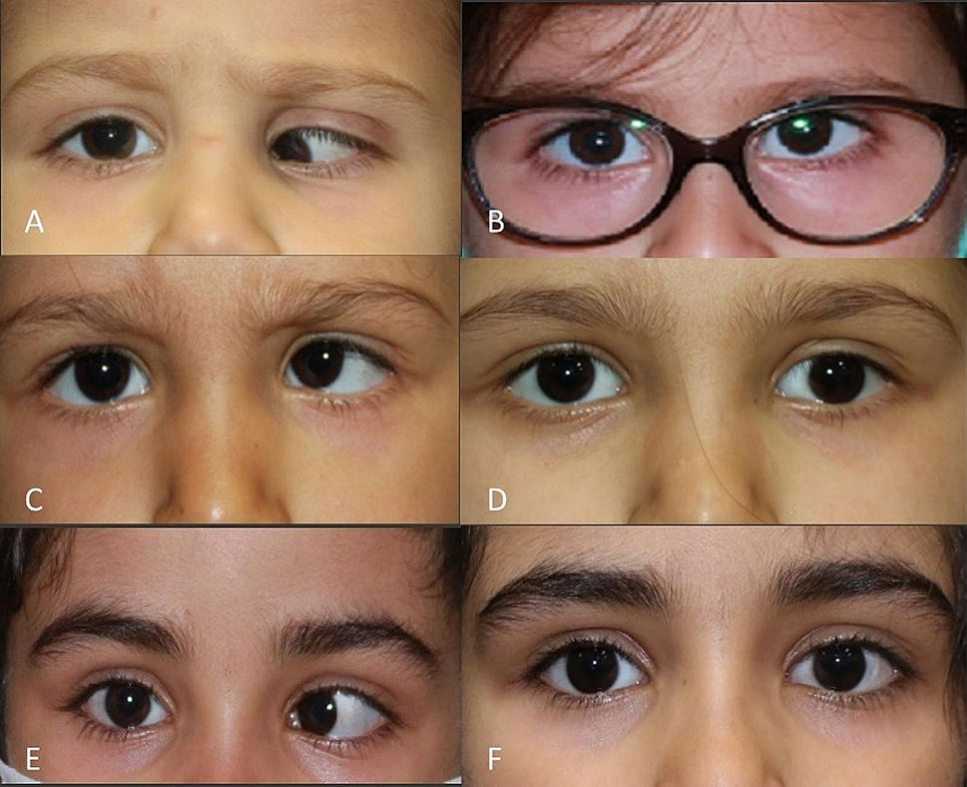
Preoperative photograph of a 4-year-old boy (no, 12) with left eye retinoblastoma, showing large angle esotropia and significant hypotropia (**A**). Six-month post strabismus surgery, photograph demonstrating small angle esotropia and acceptable result (**B**). Preoperative photograph of a 5-year-old boy (no, 16) with unilateral retinoblastoma, showing large angle esotropia and inferior oblique overaction of left eye (**C**). One year later, the photograph shows an acceptable result and slight exotropic shift (**D**). Preoperative photograph of a 6-year-old girl (no, 10) with treated left eye retinoblastoma demonstrating esotropia (**E**). Twelve months after strabismus surgery, the photograph shows orthotropia alignment (**F**)

The mean angle of deviation for esotropic patients was 42.0 ± 10.4 (range, 30–60) PD, and for exotropic patients was 35.7 ± 7.9 (range, 25–50) PD (*P* = 0.32). After surgery, the mean angle of deviation was significantly decreased to 8.5 ± 5.3 PD and 5.9 ± 6.7 PD in esotropic and exotropic cases, respectively (*P* < 0.001) (Fig. 1). The mean angle of vertical deviation before surgery was 12.4 ± 4.5 (range, 6–20) PD which was decreased significantly to 2.0 ± 1.5 (range, 0–4) PD by surgery. Patients’ strabismus features, treatments, and outcomes are listed in Table 2.

The mean follow-up period after strabismus surgery was 15.2 ± 2.0 (range, 10–24) months, that except 3 (17%), all patients were managed successfully with only one surgery. Two patients with esotropia and one patient with exotropia were overcorrected. Re-surgery of these patients was performed 4 months after the first surgery. Overcorrection was the reason for re-operation in these patients. The result of re-surgery of these patients was successful and did not require additional surgery. Occlusion therapy for amblyopic patients was continued after surgery in patients who had the potential for vision improvement. After strabismus surgery, all patients were periodically examined by an ocular oncologist. No more tumor recurrence was detected, and no patient needed more treatment regarding tumor control.

## Discussion

Eyes with advances retinoblastoma have a guarded visual prognosis and management of these patients requires a multidisciplinary approach with an ocular oncologist, pediatric oncologist, and pediatric ophthalmologist. Dramatic changes have occurred regarding Rb management in the last 30 years, including a high survival rate (almost 98% in developed countries) and significant improvements in globe salvage according to the grade of tumor burden [2, 4, 10, 11]. The main goal in treating Rb patients is to reach complete tumor control and avoid metastatic spread, save lives, save the globe, and if possible, preserve the sight of patients. Recently, new advances in the treatment of these patients have made the final goal more important.

Retinoblastoma tumors can be located in the macula and cause significant visual deprivation. In one cohort study by Fabian et al., 63% of Rb tumors were located < 1 mm from the fovea and 75% of patients had macular abnormality secondary to tumor, detached retina, and/or treatment side effects [12]. Any macular abnormality can cause visual deprivation, amblyopia, nystagmus, and strabismus.

Although the first goal in the treatment of Rb patients is to save life and prevent tumor metastasis, paying attention to the patient’s lifestyle and improving their social condition should not be ignored. Today, due to successful tumor control in most patients, we are dealing with this challenge more. Retinoblastoma is a rare disease. Therefore, extensive studies are likely to be limited.

Few studies on Rb patients have been conducted regarding strabismus in patients with salvaged globes and the prevalence and features of strabismus have mostly been mentioned descriptively [12, 13]. Strabismus has significant effects on the health-related quality of life (HRQOL) of the patients [14, 15]. People with strabismus live with more clinical anxiety or depression compared to the general population and the levels of social anxiety and social avoidance were significantly poorer than population norms [14]. Previous studies have demonstrated that HRQOL worsened in both children with strabismus and their parents, while corrective strabismus surgeries have shown significant improvement in the HRQOL scores in both children and their parents [16–18].

Apart from the reconstructive realignment of eyes and psychological benefits once the ocular alignment is restored, strabismus surgeries may be restoration of binocular single vision, binocular visual field expansion and unexpected recovery of sensory fusion [15, 16]. However, due to low vision in most involved eyes, we could not report the improvement in the sensory functions after surgery.

Intraocular surgeries in eyes with Rb remains strongly associated with challenges and the major concerns are extraocular spread and distant metastases [19, 20]. In patients with retinoblastoma, it is commonly accepted that intraocular surgeries (e.g. cataract surgery) should be delayed until complete tumor control is achieved, but there is controversy regarding the best time for doing surgery [19–20]. It is recommended to postpone these surgeries between 18 and 24 months after complete tumor control [20]. The two earliest possible safety periods are outlined as nine months by Osman et al. [21] and as early as six months by Kaliki et al. [22]. There is no consensus on the exact timing of doing strabismus surgery in patients with salvaged globe therefore, based on the previously mentioned studies, the authors decided to perform strabismus surgery 12 months after complete remission of tumors. Routine strabismus surgeries were performed for all cases and fortunately, tumor recurrence was not seen in their average follow-up of 15 months. In patients with Rb, even after the completion of the treatment period, there is a concern about the spread and recurrence of the tumor due to additional interference and surgeries. In term of strabismus surgery, hang back surgery is considered safer due to the small chance of perforation but, the low accuracy of hang back surgery and the possibility of more under and over correction caused the routine method be used [23, 24].

In a study by Fabian et al. on 42 globe-salvaged Rb patients, the prevalence of strabismus was 69% in which exotropia was the most common (*n* = 18) followed by esotropia (*n* = 8) and alternate strabismus (*n* = 5) [13]. In another study on 27 bilaterally globe-salvaged Rb patients, 12 had strabismus (*n* = 10 exotropia and *n* = 2 esotropia) [12]. In the present study on 18 patients, esotropia was more common but there was no significant difference (*p* = 0.35). Compared to previous reports, a significant number of patients showed vertical deviation (*n* = 9, 50%) and meanwhile, 4 patients (24%) underwent inferior oblique myectomy. The authors’ professional approach to strabismus and more detailed examination of patients may be the reason for this difference.

Although we could not find a similar study for comparison of surgical results but, the response of our patients to strabismus surgery was acceptable and it was close to the reports given for the outcome of surgery in general population with sensory strabismus [25, 26]. In a study by Merino et al. on patients with sensory strabismus, the final outcome was successful in 90.6% of the cases which were surgically treated [26].

Limitations of this study include its small sample size and relatively short follow-up time after surgery. After strabismus surgery, it is known that the angle of deviation may change after a certain period of time depending on the type of strabismus and duration. Despite these constraints, this study is currently the first report about strabismus surgery in Rb patients and the results of the present study shed light for the first time on strabismus surgery outcomes in Rb patients.

In conclusion, due to limited number of patients, our results will not be guiding but, the authors think the results of this study will be useful and practical for appropriate management of strabismus caused by retinoblastoma.

## Data Availability

The datasets used and analyzed during the current study are available from the corresponding author (Babak Masoomian) upon reasonable request.
